# Visual Feedback Control of a Rat Ankle Angle Using a Wirelessly Powered Two-Channel Neurostimulator

**DOI:** 10.3390/s20082210

**Published:** 2020-04-14

**Authors:** Masaru Takeuchi, Keita Watanabe, Kanta Ishihara, Taichi Miyamoto, Katsuhiro Tokutake, Sota Saeki, Tadayoshi Aoyama, Yasuhisa Hasegawa, Shigeru Kurimoto, Hitoshi Hirata

**Affiliations:** 1 Department of Micro-Nano Mechanical Science and Engineering, Nagoya University, Nagoya 4648603, Japan; watanabe@robo.mein.nagoya-u.ac.jp (K.W.); ishihara@robo.mein.nagoya-u.ac.jp (K.I.); miyamotot@robo.mein.nagoya-u.ac.jp (T.M.); tadayoshi.aoyama@mae.nagoya-u.ac.jp (T.A.); yasuhisa.hasegawa@mae.nagoya-u.ac.jp (Y.H.); 2 Department of Hand Surgery, Nagoya University, Nagoya 4668550, Japan; k.tokutake@med.nagoya-u.ac.jp (K.T.); ssaeki@med.nagoya-u.ac.jp (S.S.); kurimotos@icloud.com (S.K.); h-hirata@med.nagoya-u.ac.jp (H.H.)

**Keywords:** neurostimulation, visual feedback control, functional electrical stimulation

## Abstract

Peripheral nerve disconnections cause severe muscle atrophy and consequently, paralysis of limbs. Reinnervation of denervated muscle by transplanting motor neurons and applying Functional Electrical Stimulation (FES) onto peripheral nerves is an important procedure for preventing irreversible degeneration of muscle tissues. After the reinnervation of denervated muscles, multiple peripheral nerves should be stimulated independently to control joint motion and reconstruct functional movements of limbs by the FES. In this study, a wirelessly powered two-channel neurostimulator was developed with the purpose of applying selective FES to two peripheral nerves—the peroneal nerve and the tibial nerve in a rat. The neurostimulator was designed in such a way that power could be supplied wirelessly, from a transmitter coil to a receiver coil. The receiver coil was connected, in turn, to the peroneal and tibial nerves in the rat. The receiver circuit had a low pass filter to allow detection of the frequency of the transmitter signal. The stimulation of the nerves was switched according to the frequency of the transmitter signal. Dorsal/plantar flexion of the rat ankle joint was selectively induced by the developed neurostimulator. The rat ankle joint angle was controlled by changing the stimulation electrode and the stimulation current, based on the Proportional Integral (PI) control method using a visual feedback control system. This study was aimed at controlling the leg motion by stimulating the peripheral nerves using the neurostimulator.

## 1. Introduction

Recently, implantable devices for functional electrical stimulation (FES) has been actively developed and applied to reconstruct functional motions of patients who lost their functional movements of limbs by a spinal cord injury. For example, Ajiboye et al. developed the FES system with Brain-Computer Interface (BCI) to regain limb movements through FES of peripheral muscles and nerves [1]. Bouton also developed neuromuscular electrical stimulation sleeve to restore movements of limbs in a paralyzed human [2]. In these studies, the muscles could respond to the FES after the spinal injury because the lower motor neurons still maintained excitability to the electrical stimulation. Thus, the FES has been applied to restore functional motion for patients with spinal cord injury.

On the other hand, peripheral nerve disconnections caused by traffic accidents such as brachial plexus injury or neurodegenerative disease of peripheral nerves such as Amyotrophic lateral sclerosis (ALS) cause severe muscle atrophy and consequently paralysis of limbs. The lower motor neurons lose the excitability to the electrical stimulation in such peripheral nerve disconnections. Therefore, an irreversible degeneration of muscle tissues has to be prevented to retain muscle function after the occurrence of peripheral nerve disconnections. The transplantation of motor neurons onto peripheral nerves with the FES treatment is one approach for preventing the degeneration of denervated muscles. Recovery of muscle activity has previously been seen to occur following electrical stimulation of the peroneal or tibial nerve after injection of motor neurons into the sciatic nerve of a rat [3,4]. The application of FES has demonstrated its potential for slowing down the atrophy of denervated muscles and allowing the retention of muscle functionality [5].

In a previous study, the angle of an ankle joint was found to increase during walking motion of a rat using FES on a peripheral nerve that had been subjected to a procedure that produces Motoneuron Integrated Striated Muscle (MISM) [3,4]. MISM is the outcome of a procedure whereby motor neurons are transplanted into a peripheral nerve to restore muscle functionality. In the previous study, FES was applied to the nerve via a conductive wire connected to a power source located outside of the rat body. Such a wired nerve stimulation system generates infection risk and the inhibition of motion of the rat. Therefore, an implantable wireless microelectrode for nerve stimulation is desirable.

The implantable FES systems to stimulate peripheral nerves have been developed, and some devices have been clinically applied to actual patients such as Vagus Nerve Stimulation (VNS) for treatment of epilepsy and artificial cochlea [6,7,8,9]. Some implantable FES devices have a function of feedback control, such as Deep Brain Stimulation systems [10,11,12] and VNS systems [13,14]. Generally, the VNS systems use one electrode to modify the stimulation signal using the closed-loop control. However, to reconstruct the functional motion of limbs, the present posture of limbs should be measured, and the multiple FES signal should be applied to multiple peripheral nerves based on the measured information because one joint motion is generally controlled by more than two muscles: the agonist muscle and the antagonist muscle. The multiple stimulation sites should conduct nerve stimulation control independently based on the feedback control. The DBS systems have such multiple electrodes, and the stimulation sites are modified by the feedback signal. In this study, we applied multiple stimulations with feedback control to achieve control of limb motion. In addition, the receiver device in our system did not have a battery, and the power activating the receiver device was completely supplied from the transmitter device. The stimulation sites and stimulation strength were dynamically controlled by using the frequency and amplitude of the transmitter signal. Thus, our system applied feedback control of multiple stimulation sites for controlling dynamically movable limbs using the battery-less simple structure.

**Figure 1:**
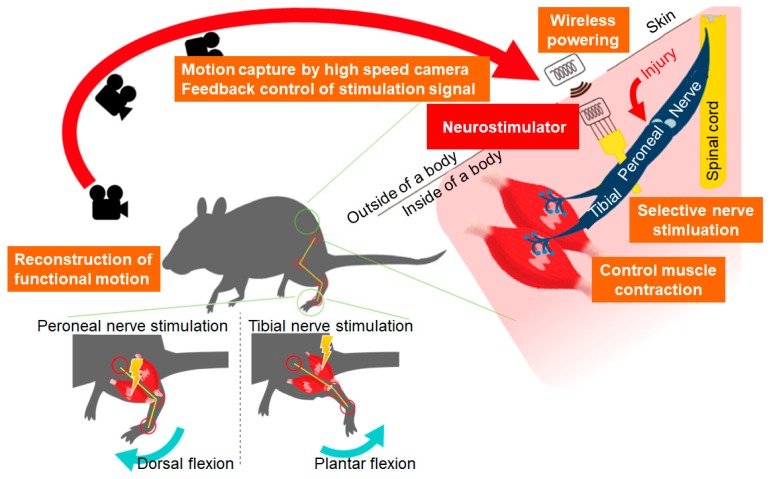
A schematic representing the concept of selective stimulation of rat peroneal and tibial nerves using visual feedback control: peroneal and tibial nerve stimulation was switched to control leg motion based on the motion capture of a rat leg by a high-speed camera.

To achieve such feedback control to modify the FES signal to multiple peripheral nerves, a new 2-channel neurostimulator was prepared, such that two peripheral nerves could be stimulated for the present study. The neurostimulator was developed based on a nerve stimulation device that was developed in our previous study that can stimulate a rat peroneal nerve using wireless powering by a magnetic resonance method [15]. The two-channel neurostimulator developed in this study allowed the actuation of both agonist and antagonist muscles by the neurostimulator, such that the ankle angle of a rat could be controlled using a visual feedback system as shown in Figure 1.

## 2. Wirelessly Powered Neurostimulator

### 2.1. Receiver System for Selective Stimulation of Two Peripheral Nerves

**Figure 2:**
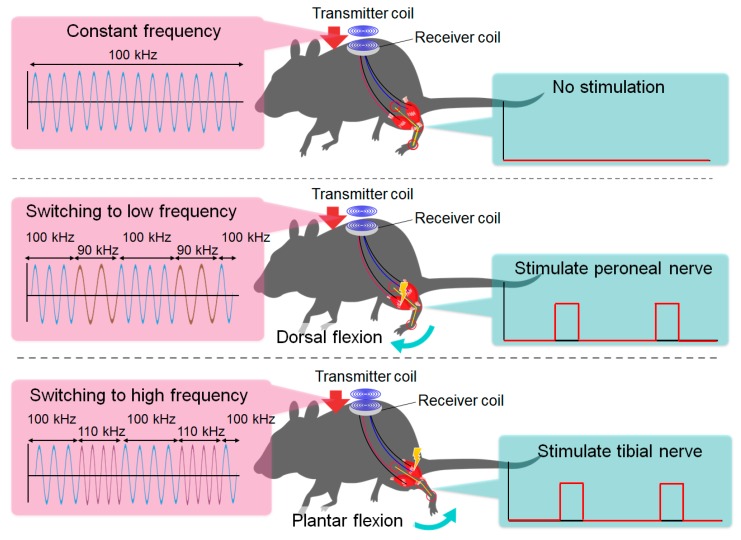
A schematic indicating the selective stimulation of the rat peroneal and tibial nerves by switching the transmitter frequency.

In this study, a wirelessly powered neurostimulator was developed to selectively stimulate two nerves in a rat, namely the tibial and peroneal nerves, to control the motion of the ankle joint. The wireless power supply was achieved by a magnetic resonance method [16,17]. A schematic of the selective electrical stimulation of peripheral nerves to control ankle joint flexion by the wirelessly powered neurostimulator is shown in Figure 2. The switching of the nerve stimulation was enabled by switching the frequency of the transmitter signal providing wireless power. In our device, three different frequencies, namely 90 kHz, 100 kHz, and 110 kHz, were used to transmit power from a transmitter device to a receiver device by a magnetic resonance method. The receiver device stimulated the peroneal nerve to generate dorsal flexion when 90 kHz was used for the wireless powering, while it stimulated the tibial nerve to generate plantar flexion when 110 kHz was used. A frequency of 100 kHz was used to stimulate neither the peroneal nerve nor the tibial nerve. Thus, the nerve to be stimulated was selected by switching the frequency of the transmitter signal. The stimulation current was controlled by the amplitude of the transmitter signal. The stimulation frequency, duration, and current were all controlled by the proposed method.

**Figure 3:**
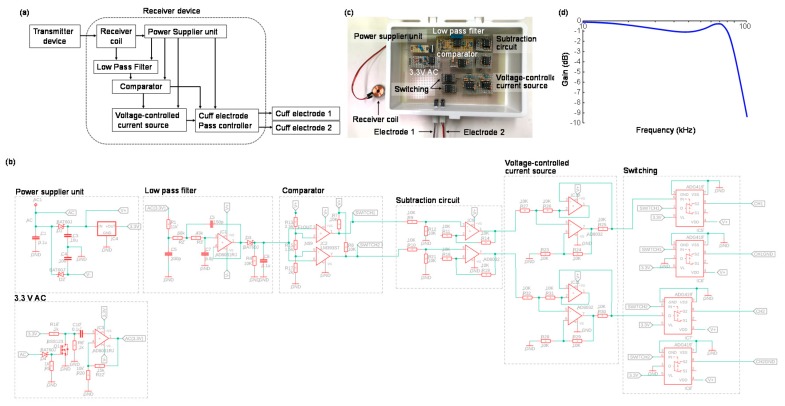
A receiver device system for visual feedback control of the rat ankle angle: (**a**) A schematic of the receiver device system; (**b**) The electrical circuit of the receiver device; (**c**) The fabricated receiver device; (**d**) The bode diagram of the low pass filter to identify the transmitter frequency.

The schematic of the receiver system of our neurostimulator is shown in Figure 3a. The system has four main functions: 1. Receive power from a transmitter device; 2. Use a low pass filter and comparator to identify the frequency of the transmitter signal; 3. Implement voltage-current translation to achieve constant current control; 4. Switch the stimulation electrodes for selective nerve stimulation. The receiver device had two stimulation electrodes, and the stimulation electrode and stimulation current were selected from the transmitter signal through the receiver system. The cuff electrode of stimulation was determined by the frequency with which the transmitter frequency was switched, and the duration of the stimulation was fixed by the duration of the transmitter signal, at either 90 kHz or 110 kHz. The receiver coil, which was used in the receiver system, had 18 mm in diameter, 26 μF self-inductance, and quality factor 25. The electrical circuit of the receiver device and fabricated receiver device are presented in Figure 3b,c. The 3rd order Sallen–Key low pass filter with the cutoff frequency at 90 kHz was used to identify the frequency of the transmitter signal. The Bode diagram of this low pass filter is shown in Figure 3d. The transmitter frequency could be determined by the filter, and the stimulation electrode was selected. In this study, the receiver device was not fabricated in implantable size to validate the stimulation signal from the receiver device could control planter/dorsal flexion of a rat ankle joint. The device will be miniaturized in the implantable size by the surface-mount package of the electrical circuit.

### 2.2. The Transmitter System for Visual Feedback Control

**Figure 4:**
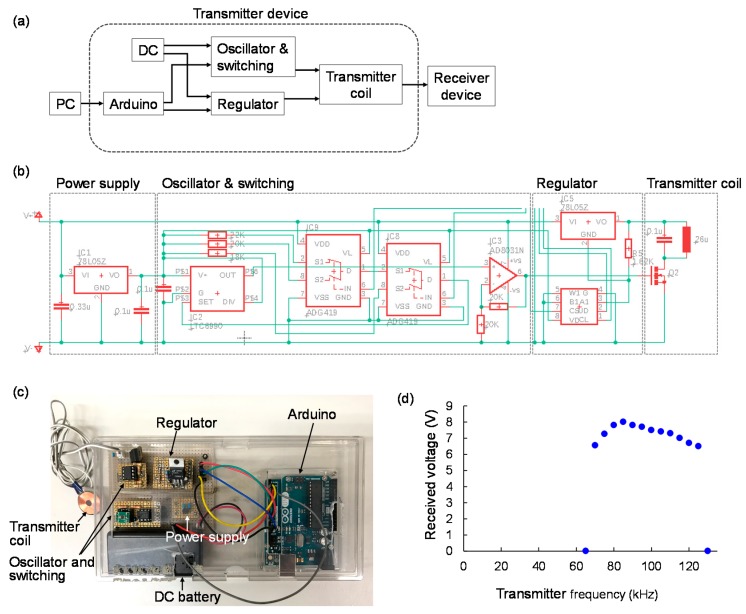
A transmitter device system for visual feedback control of the rat ankle angle: (**a**) A schematic of the transmitter device system; (**b**) The electrical circuit of the transmitter device; (**c**) The fabricated transmitter device; (**d**) The relationship between the transmitter frequency and received voltage on the receiver device.

In this study, a rat ankle angle was controlled using a visual feedback control system. The transmitter system for visual feedback control of the rat ankle angle was prepared, as shown in Figure 4. The transmitter device was composed of three parts with different functions: 1. A microcomputer to receive information about nerve stimulation and current level from a PC; 2. An oscillator to control the transmitter frequency based on the output from the microcomputer; 3. A voltage regulator to adjust the stimulation current based on the output from the microcomputer, as shown in Figure 4a. The transmitter device sent information about which nerve should be stimulated and how much stimulation current was required to the receiver device by integrating these functions. Figure 4b shows the design of electric circuit for the transmitter device, and Figure 4c indicates the fabricated transmitter device in this study. The transmitter coil used in the transmitter system was the same as the receiver coil (18 mm in diameter, 26 μF self-inductance, and 25 quality factor).

The wireless powering in different transmitter frequencies was tested using the fabricated transmitter and receiver systems. Figure 4d indicates the rectified/smoothed received voltage of the receiver device in different transmitter frequencies. Hence, the wireless powering system could send power to the receiver deice when the transmitter frequency was between 70 kHz and 125 kHz.

### 2.3. The Cuff Electrode for the Nerve-Neurostimulator Interface

Peripheral nerve electrodes are typically divided into extrafascicular electrodes and intrafascicular electrodes [18]. Intrafascicular electrodes can achieve high selectivity by placing the electrodes within the fascicles of the nerve. However, nerve damage and long-term stability are concerns when using these electrodes. By contrast, extrafascicular electrodes like cuff electrodes have attracted a substantial amount of attention due to their noninvasive nature.

**Figure 5:**
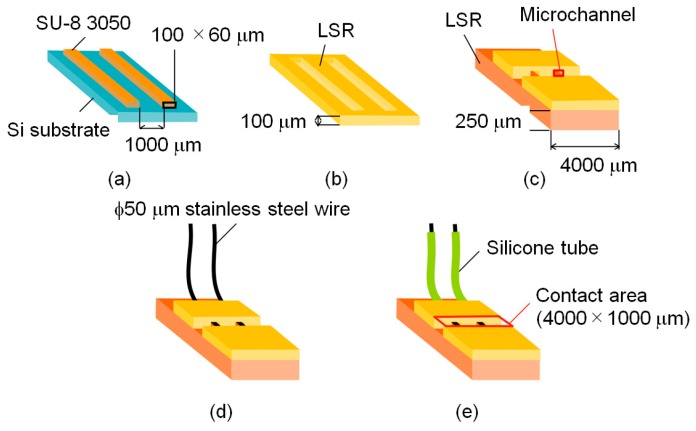
Fabrication procedure of the cuff electrode. The sequence from (**a**–**e**) indicates the sequential assembly steps of the electrode.

**Figure 6:**
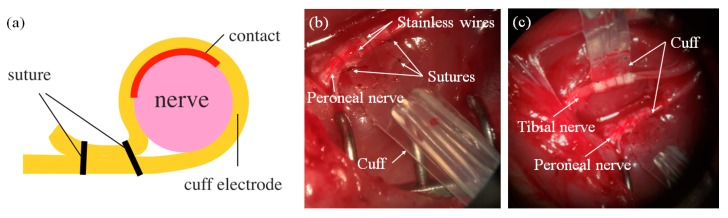
Cuff electrode as a neurostimulator-nerve interface: (**a**) A cross-sectional view of the cuff electrode fixed on a peripheral nerve; (**b**) The actual cuff electrode fixed on a rat peroneal nerve; (**c**) The two cuff electrodes fixed on a rat peroneal and tibial nerve respectively.

In this study, we developed a cuff electrode made of liquid silicone rubber (LSR) (Ecoflex 00-30 (Smooth On, Inc., Macungie, PA, USA)), which had Young’s modulus at 0.125 MPa [19,20,21] and stainless-steel wire of 50 μm in diameter. The fabrication procedure is illustrated in Figure 5. Firstly, an epoxy-based photoresist (SU8 3050 (Microchem, Inc., Westborough, MA, USA)) was patterned on a silicon substrate, as shown in Figure 5a. A solid rectangular structure of 100 μm width and 60 μm height was fabricated by a general photolithography process. This structure was transcribed to the 100 μm thick LSR film (Figure 5b). This film was cut and adhered to a 250 μm thick LSR film to make microchannels (Figure 5c). Two stainless-steel wires with diameters of 50 μm were inserted into the microchannels (Figure 5d). Finally, these wires were isolated with silicone tubing and connected to the LSR films with microchannels (Figure 5e). In experiments using a rat, the fabricated cuff electrodes were placed on a tibial nerve and a peroneal nerve, respectively. Cuff electrodes were wrapped around the nerves with sutures (Figure 6a) to get stable contacts between the nerve surfaces and the stainless-steel wires. A photograph of the fabricated cuff electrode placed on a rat peroneal nerve is shown in Figure 6b,c indicates the connection of two cuff electrodes on a rat peroneal and tibial nerve, respectively.

## 3. Evaluation of the Fabricated Neurostimulator

### 3.1. Relationship Between the Stimulation Current and the Gap between the Transmitter/Receiver Coils

The gap between the transmitter coil and the receiver coil is an important parameter for wireless powering by the magnetic resonance method. In general applications, the receiver device will be implanted inside a rat body, and the gap between the two coils will not be directly measured and precisely fixed. Therefore, the wireless powering should be achieved even if that gap varies by a few millimeters. In the present study, the output of the receiver device was connected to a resistance of 1 kΩ, which simulated the resistance of a peripheral nerve. The maximum power was sent wirelessly from the transmitter device to the receiver device, and the applied voltage over the 1 kΩ resistance was measured. Combining this measured voltage and the resistance of 1 kΩ, the output current from the receiver device could be calculated. The change in the output current from the receiver device was checked as the gap was changed from 1 mm to 5 mm. Glass slides were placed between the transmitter and receiver coils to make the gap. The effect of the difference in vivo and in vitro for the coupling coefficient of two coils could be ignored because the relative permeability of glass is 1, while the water is 0.999991.

**Figure 7:**
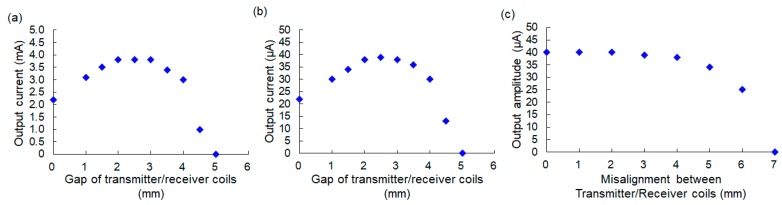
Experimental results for the output current from the receiver device: (**a**) Results with varying values of the vertical gap between the transmitter coil and the receiver coil when the output current was set on the maximum value in the experimental setup; (**b**) Results with varying values of the vertical gap between the transmitter coil and the receiver coil when the output current was set on μA order for visual feedback experiments using a rat; (**c**) Results with varying values of the horizontal misalignment between the transmitter coil and the receiver coil.

The experimental results for the relationship between the vertical gap separating the transmitter and receiver coils and the output current from the receiver device (simulating peripheral nerves) are indicated in Figure 7a when the output current was set on the maximum current in our experimental setup. Generally, a rat’s skin thickness is around 1 mm, so the coil gap becomes larger than 1 mm when the receiver device is implanted inside a body. The experimental results show that the stimulation current remained higher than 3 mA when the coil gap was between 1 mm and 4 mm. A current of 3 mA was large enough to generate full dorsal/plantar flexion of a rat ankle, as shown in the visual feedback experiments explained in Section 4 of this paper.

In the experiments explained in Section 4, we used the stimulation current at μA order. Therefore, the output current was set on μA order (the same setup used in the experiments using rats in Section 4), and the output current was checked using the same procedure of the experiment, as shown in Figure 7a. Figure 7b showed the results when the vertical gap was changed. Thus, the same characteristics were obtained even if the output current was set on the μA order, and the output current was not changed dramatically if the coil gap was between 1 mm and 4 mm. The output current was also measured when the horizontal misalignment between the transmitter and receiver coils was changed. The experiment was conducted by keeping the vertical gap at 2.5 mm and change the position of the transmitter coil horizontally. The output current started to decrease from 3 mm misalignment, and it became about 62% of maximum current when the misalignment became 4 mm, as shown in Figure 7c. The output current was dropped 25% of the maximum current when the misalignment became 5 mm. Hence, the result indicates that the wireless power system that was developed for this study was capable of sending sufficient power to stimulate peripheral nerves, even if the embedded receiver coil was moved by a few millimeters inside a rat body after implantation.

### 3.2. The Ability of Switching to Stimulate Two Different Nerves

Switching tests were conducted to check how quickly stimulations could be switched in our device. When the device is applied to the reconstruction of the functional motion of limbs, it should be possible to stimulate currents in different electrodes independently. Therefore, switching tests are important. To check the switching ability of the device, the output currents were set to the maximum output value (3.6 mA) of our neurostimulator on one electrode and to the minimum output value (2.2 mA) on the other electrode, with a coil gap of 2.5 mm at first.

**Figure 8:**
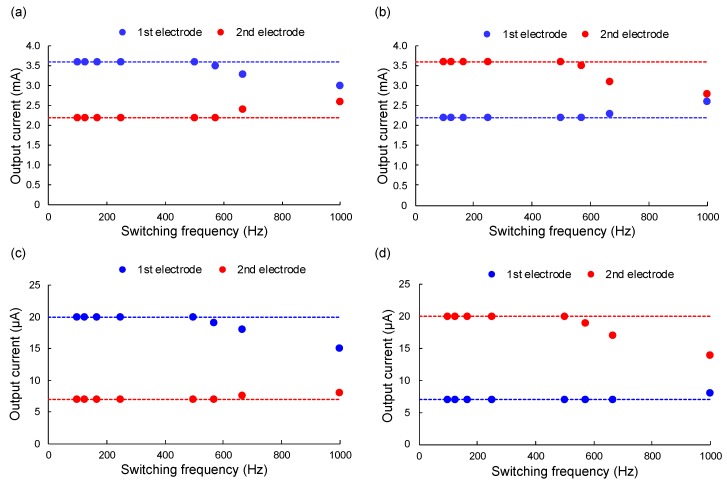
Experimental results for the switching of two electrodes for different frequencies: (**a**) When the output currents in the 1st and 2nd electrodes were set to 3.6 mA and 2.2 mA, respectively; (**b**) When Table 1st and 2nd electrodes were set to 2.2 mA and 3.6 mA, respectively; (**c**) When the output currents in the 1st and 2nd electrodes were set to 20 μA and 7 μA, respectively; (**d**) When the output currents in the 1st and 2nd electrodes were set to 7 μA and 20 μA, respectively. Dashed lines indicate the target output current in each electrode.

The experimental results are shown in Figure 8a,b. At first, the target output currents in the first and second electrodes were set to 3.6 mA and 2.2 mA, respectively. As shown in Figure 8a, the outputs from both electrodes drifted away from the target currents (and started approaching the same value) at switching frequencies above 600 Hz, while the outputs from both electrodes remained equal to the target currents at switching frequencies below 500 Hz. Similar results were obtained when the target output currents in the 1st and 2nd electrodes were set to 2.2 mA and 3.6 mA, respectively, as shown in Figure 8b.

The same experiments were conducted after reducing the maximum output value (20 μA) and the minimum output value (7 μA) in the experimental setup. This stimulation range was used for the visual feedback experiments using rats explained in Section 4. The experimental results are shown in Figure 8c,d. Thus, the outputs from both electrodes drifted away from the target currents (and started approaching the same value) at switching frequencies above 600 Hz, while the outputs from both electrodes remained equal to the target currents at switching frequencies below 500 Hz. Hence, the results indicate that the developed device could control output currents from two electrodes independently if the switching frequency remained below 500 Hz.

### 3.3. The Selective Stimulation of the Peroneal and Tibial Nerves

**Figure 9:**
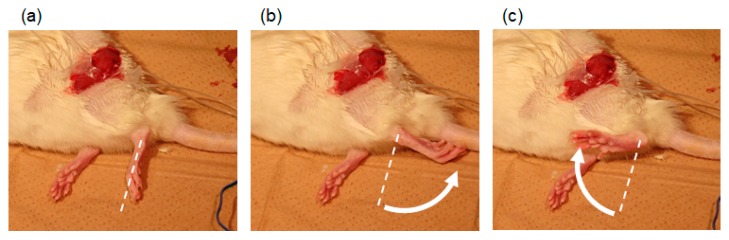
Selective stimulation of rat peroneal and tibial nerves (**a**) Without stimulation; (**b**) With stimulation to the tibial nerve to generate plantar flexion of the ankle joint; (**c**) With stimulation to the peroneal nerve to generate dorsal flexion of the ankle joint.

The cuff electrodes were connected to the rat peroneal and tibial nerves to generate dorsal and plantar flexion of the ankle joint, respectively. In the experiment, the rat was under anesthesia, and the stimulation signal at 50 Hz, 0.2 ms duration was used. The stimulation current of the tibial nerve was set on 0.40 mA, and that of peroneal nerve was 0.40 mA to check the flexion of the ankle joint. In the experiments, the receiver device was placed outside the body, and the two outputs from the receiver device were connected to the two cuff electrodes fixed on the peroneal and tibial nerves. The experimental results are shown in Figure 9. Plantar flexion (Figure 9b) and dorsal flexion (Figure 9c) were generated by the stimulation of the tibial nerve and the peroneal nerve, respectively. Hence, the results indicate that the developed device can generate dorsal and plantar flexion of a rat ankle joint, by design.

## 4. Visual Feedback Control of the Rat Ankle Joint

### 4.1. The Experimental Setup for Visual Feedback Control of the Rat Ankle Joint

**Figure 10:**
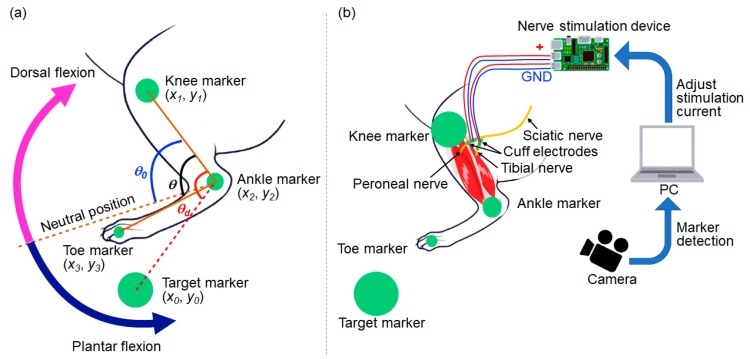
Experimental setup for visual feedback control of the rat ankle angle: (**a**) A schematic of the markers placed on a rat leg for detecting the ankle angle and the target angle; (**b**) A schematic of the experimental system for visual feedback control.

In order to achieve feedback control of the rat ankle joint angle, the current position of the rat leg has to be detected. In our experiment, a camera (C-ST, Photron, Tokyo, Japan) was used to detect the angle of the ankle joint. Three green markers were placed on the rat toe, ankle, and knee, respectively. The experimental setup for calculating the ankle joint angle from the markers is shown in Figure 10a. In the visual feedback experiments, the green markers placed on the rat hind leg were detected using the parameters about the color space of the Hue, Saturation, Value (HSV) model. In our experimental setup, each parameter had a number from 0 to 255, and the four threshold values: minimum Hue, maximum Hue, Saturation, and Value were set at the start of experiments. At first, the threshold of minimum Hue was gradually increased to detect the markers on the rat hind leg in our experimental setup. The threshold of maximum Hue, Saturation, and Value were fixed in sequence by increasing the threshold to detect the markers. Each parameter was set as shown below: the minimum Hue was 91, the maximum Hue was 128, Saturation was 45, and Value was 29 in the experiments.

The different sizes of markers were used to identify the body part of each marker. The largest marker was used for the target marker, and markers on the knee, ankle, and toe became smaller in this order. From the placement of these markers on the rat leg, the knee position (*x*_1_, *y*_1_), ankle position (*x*_2_, *y*_2_), and toe position (*x*_3_, *y*_3_) were detected. The angle of the ankle joint *θ* was calculated using Equation (1):

(1) $$\theta =\arccos\frac{\left(x_{1}-x_{2}\right)\left(x_{3}-x_{2}\right)+\left(y_{1}-y_{2}\right)\left(y_{3}-y_{2}\right)}{\sqrt{{\left(x_{1}-x_{2}\right)}^{2}+{\left(y_{1}-y_{2}\right)}^{2}}\sqrt{{\left(x_{3}-x_{2}\right)}^{2}+{\left(y_{3}-y_{2}\right)}^{2}}}\;$$

The target angle *θ_d_* was calculated using the target marker position (*x*_0_, *y*_0_) in Equation (2):

(2) $$\theta_{d}=\arccos\frac{\left(x_{1}-x_{2}\right)\left(x_{0}-x_{2}\right)+\left(y_{1}-y_{2}\right)\left(y_{0}-y_{2}\right)}{\sqrt{{\left(x_{1}-x_{2}\right)}^{2}+{\left(y_{1}-y_{2}\right)}^{2}}\sqrt{{\left(x_{0}-x_{2}\right)}^{2}+{\left(y_{0}-y_{2}\right)}^{2}}}\;$$

The stimulation current received by the tibial or peroneal nerve was adjusted using the Proportional Integral (PI) control method to change *θ* so that it was equal to *θ_d_*. *θ*_0_ is the neutral angle of the rat ankle joint. The plantar flexion can be generated by stimulating the tibial nerve, and the dorsal flexion can be generated by stimulating the peroneal nerve. The angle *θ* becomes larger than *θ_0_* during the plantar flexion while *θ* becomes smaller than *θ*_0_ during the dorsal flexion.

In order to generate dorsal flexion (the case where $\theta_{d}<\theta_{0}$), the stimulation current to the peroneal nerve at the time step *k* (represented by *S^p^(k)*) was adjusted using Equations (3)–(5), as shown below:

(3) $$S_{p}^{p}\left(k\right)=K_{p}\left(\theta -\theta_{d}\right);$$

(4) $$S_{i}^{p}\left(k\right)=S_{i}\left(k-1\right)+K_{i}\left(\theta -\theta_{d}\right);$$

(5) $$S^{p}\left(k\right)=S_{p}^{p}\left(k\right)+S_{i}^{p}\left(k\right),$$

where $S_{p}^{p}\left(k\right)$ is the stimulation current at time step *k* calculated by Proportional (P) control, and $S_{i}^{p}\left(k\right)$ is the stimulation current at time step *k* calculated by Integral (I) control, for the case where $\theta_{d}<\theta_{0}$. *K_p_* and *K_i_* are constant coefficients of P control and I control, respectively.

On the other hand, in order to generate the plantar flexion (the case where $\theta_{d}\geq \theta_{0}$), the stimulation current to the tibial nerve at the time step *k* (represented by *S^t^(k)*) was adjusted using Equations (6)–(8), as shown below:

(6) $$S_{p}^{t}\left(k\right)=K_{p}\left(\theta_{d}-\theta \right);$$

(7) $$S_{i}^{t}\left(k\right)=S_{i}\left(k-1\right)+K_{i}\left(\theta_{d}-\theta \right);$$

(8) $$S^{t}\left(k\right)=S_{p}^{t}\left(k\right)+S_{i}^{t}\left(k\right),$$

where $S_{p}^{t}\left(k\right)$ is the stimulation current at time step *k* calculated by P control, and $S_{i}^{t}\left(k\right)$ is the stimulation current at time step *k* calculated by I control, for the case where $\theta_{d}\geq \theta_{0}$.

The experimental setup for visual feedback control of the ankle angle is shown in Figure 10b. The rat was under anesthesia during the experiment. The two cuff electrodes were connected to the tibial and peroneal nerves, respectively, as shown in Figure 6c, and the stimulation current, which was applied from the stimulation device generated either plantar flexion or dorsal flexion of the ankle. The sampling frequency of the high-speed camera was 250 Hz, and the frequency of application of the stimulation current was controlled at 12 Hz in our visual feedback system. The control frequency was restricted by the serial communication speed from PC to the microcomputer (Arduino) in our experimental setup. The transmitter system used 0.25 A, 9.0 V, 2.25 W with the coil gap 2.2 mm to activate the receiver system by wireless powering in the experiments.

To determine the current range for FES, the stimulation current was gradually increased before the visual feedback experiments. The minimum stimulation current of perineal nerve$\;S_{min}^{p}$ was fixed when the ankle leg was started to respond to the stimulation, and the maximum stimulation current of perineal nerve $S_{max}^{p}$ was fixed when the ankle leg became maximum flexion angle. The same procedures were conducted to determine the minimum and maximum stimulation current for tibial nerve $S_{min}^{t}$ *and* $S_{max}^{t}$*,* respectively.

After determined the range of stimulation currents, the visual feedback experiments were conducted. At first, constant coefficients of I control *Ki* was set on 0, and the constant coefficients of P control *K_p_* was gradually increased, then it was fixed at the value just before the vibration of rat leg was observed. Thereafter, *K_i_* was gradually increased, and it was also fixed at the value just before the vibration of the rat leg was observed.

### 4.2. Step Response of the Rat Ankle Joint

The step response of the rat ankle joint was evaluated by stimulating either the tibial or the peroneal nerve. In these experiments, the ankle angle was measured when the nerves were stimulated. The neutral position (for no stimulation of the nerves) corresponded to an ankle angle of 80°. The parameters for PI control was determined by the procedure described in Section 4.1. The transmitter system used 0.25 A, 9.0 V, 2.25 W with the coil gap 2.2 mm for the wireless powering. The parameters for PI control were set to the values *K_p_* = 5 and *K_i_* = 0.2. The target angle of the ankle joint took three successive values: 40°, 50°, 60°, and each response of the rat ankle angle was measured in sequence. The rat was under anesthesia during the experiments.

**Figure 11:**
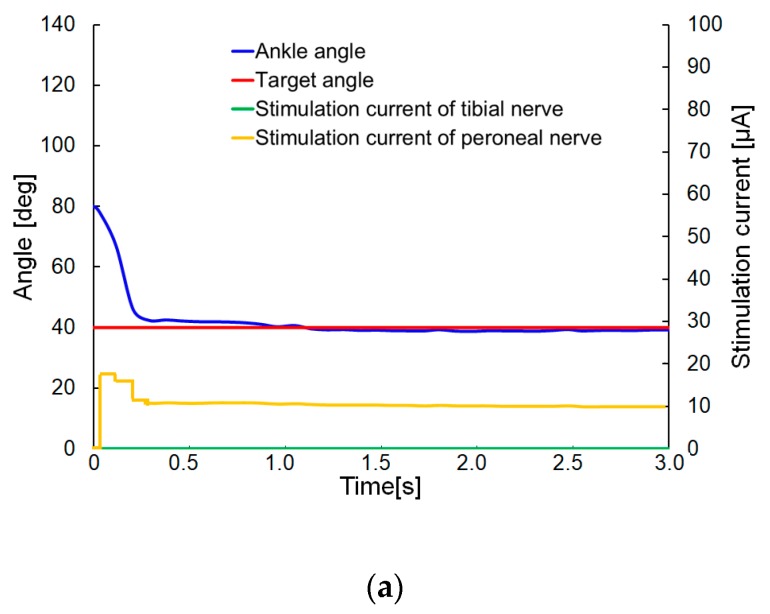

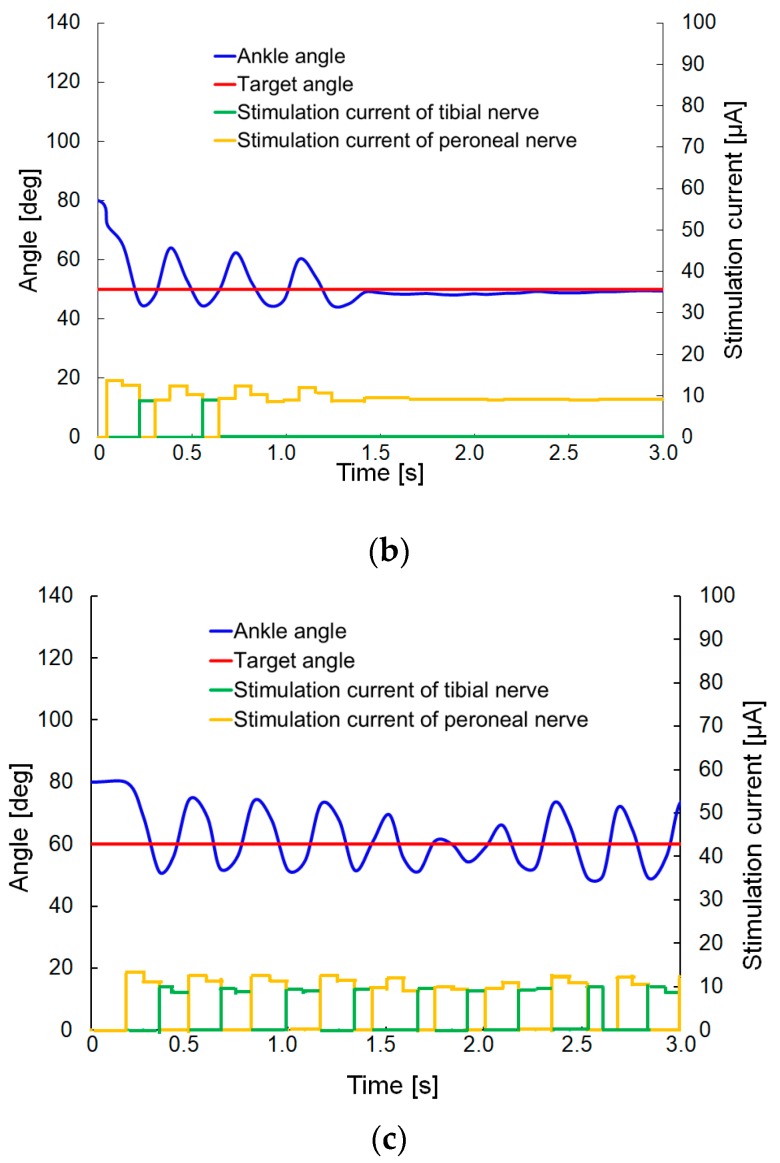
Experimental results for the step response: Results are shown for the following target angles for the rat ankle joint: (**a**) 40°; (**b**) 50°; (**c**) 60°.

Figure 11 shows the experimental results (supplementary video S1 contained movies of the step response when the target angles were 40 and 50°). When the target value for the rat ankle angle was set to 40°, the target angle reached to approximately 0.2 ms, without overshoot, as shown in Figure 11a. In the case when the target value was set to 50°, the ankle angle was overshot. After some initial fluctuation of the target angle, the ankle angle eventually settled on the target value after approximately 1.4 s, as shown in Figure 11b. In the case when the target angle was 60°, the ankle angle also overshot the target angle and continued oscillating about the target angle, as shown in Figure 11c. In other words, an oscillation of the ankle joint was generated when the target angle was close to the angle corresponding to the neutral position. These results may be ascribed to a nonlinear response of the dorsal/plantar flexion angles to the stimulation current. When the ankle angle was close to the neutral position, a small current increase caused a large change in ankle angle, while a larger current increase was needed to obtain the same angle change when the ankle angle was close to the maximum flexion angle [21]. When the target angle was far from the neutral position (for instance, when the target angle was 40°, as shown in Figure 11a), the initial stimulation current flowing into the peroneal nerve was not sufficient to cause overshoot of the target angle. The time response to the target angle 40° was 0.2 s. On the contrary, when the target angle was close to the neutral position (for instance, when the target angle was 60°), the initial stimulation current flowing into the peroneal nerve was sufficient to cause an overshoot of the target angle, as shown in Figure 11c). Once the overshooting had occurred, and the stimulation was switched from the peroneal nerve to the tibial nerve, an oscillation of the leg was generated, as shown in Figure 11b,c, and the oscillation could not be stopped once the target angle had approached the angle of the neutral position too closely, as shown in Figure 11c. The time response to the target angle 50° was 1.4 s.

### 4.3. Visual Feedback Control of the Rat Ankle Joint

Visual feedback control of the rat ankle joint was conducted with our developed system. In that experiment, the target marker was moved manually, and the stimulation currents to the peroneal and tibial nerves were controlled by the PI control method. The parameters for PI control were initially set to values of *K_p_* = 5 and *K_i_* = 0.2. The transmitter system used the same condition of the step response (0.25 A, 9.0 V, 2.25 W with the coil gap 2.2 mm) for the wireless powering. The rat was under anesthesia during the experiment.

**Figure 12:**
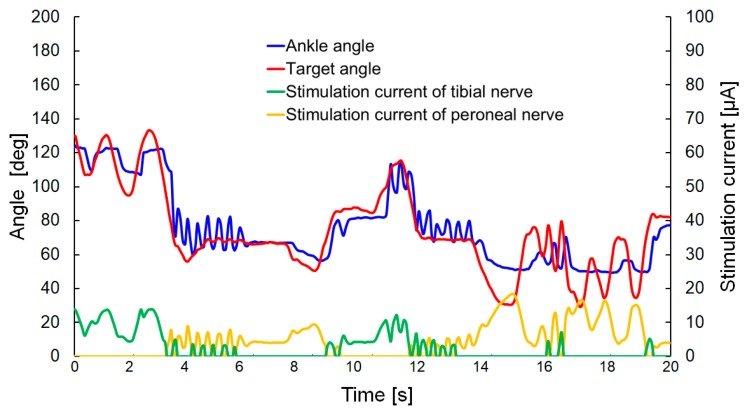
Experimental results for visual feedback control of the rat ankle joint when *K_p_* = 5; *K_i_* = 0.2.

The experimental results are shown in Figure 12 (the supplementary video S1 contains movies of the visual feedback control). The ankle angle followed the target angle without a large time delay, but the oscillation of the leg was observed when the target angle approached the neutral position (corresponding to an angle of approximately 80°). This outcome was similar to the result observed in the step response for target angles of 40 and 50°.

In order to decrease the oscillation occurring when the ankle angle approached the neutral position, the P control method was implemented with a change from Equations (3) and (6) to the equations shown below:

(9) $$S_{p}^{p}\left(k\right)=K_{p}\left(\theta_{0}-\theta_{d}\right)\;(\mathrm{if}\;\theta_{d}<\theta_{0});$$

(10) $$S_{p}^{t}\left(k\right)=K_{p}\left(\theta_{d}-\theta_{0}\right)\;(\mathrm{if}\;\theta_{d}\geq \theta_{0}).$$

No change was applied for the implementation of the I control method, described by Equations (4) and (7). In that method, the stimulation of P control was proportional to the difference between the neutral position *θ_0_* and the target angle *θ_d_*, and not affected by the ankle angle *θ*. By adjusting the parameter *K_p_*, the oscillation could be decreased effectively.

**Figure 13:**
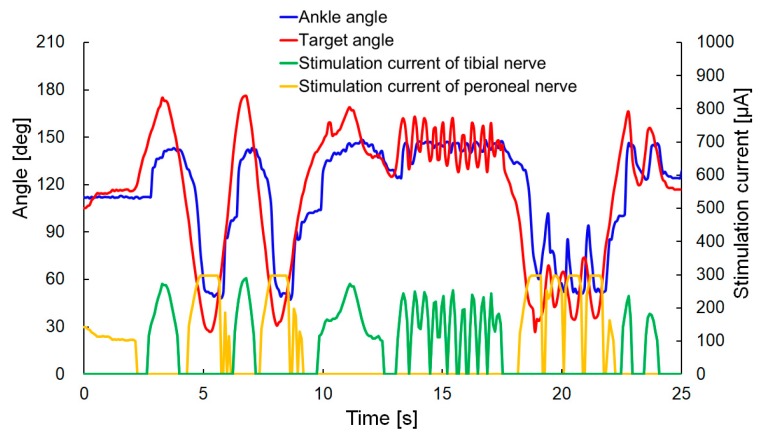
Experimental results of visual feedback control of rat ankle joint when *K_p_* = 1 and *K_i_* = 0.02.

The experimental results for the values *K_p_* = 1 and *K_i_* = 0.02 are shown in Figure 13 (the supplementary video S1 contains the movies of the visual feedback control). The rat was under anesthesia during the experiment. In that experiment, the stimulation current had a higher value than in the previous experiment (as shown in Figure 12) because the experiment was conducted on a different rat and the varying quality of the electrical contact with the cuff electrode led to a variation in the current required to generate dorsal and plantar flexion. The overshoot and oscillation of the leg were not generated in that experiment. However, the response of the ankle angle was decreased, and the steady-state error was increased, compared with the previous experiment. These results may be due to the smaller value of *K_i_* and the fact that the stimulation based on P control was not influenced by the ankle angle *θ*. The degree of control of the ankle angle will be improved by combining the two approaches used for visual feedback control (with the respective results shown in Figure 12 and Figure 13), in order to improve the response time and reduce the steady-state error without generating an oscillation in the rat leg.

## 5. Discussion

In this study, we applied FES to peripheral nerves to generate leg motion. In general, FES can be divided into three types [22]. First, there is surface stimulation [23]. In this version of FES, the electrodes for stimulation are placed on a layer of skin over the target nerves or motor points of muscles to be activated. Surface stimulation is non-invasive but can be uncomfortable or painful during stimulation. Second, there is percutaneous stimulation. The electrodes are implanted into the muscles to be activated. Percutaneous stimulation has excellent muscle selectivity but requires a lot of power for stimulation and has a risk of infection [24]. The third type of FES is an implanted neuroprosthetic stimulation [25,26,27,28]. The electrodes are implanted on the target nerve. Implanted neuroprosthetic stimulation requires only one-tenth of the amount of power required by percutaneous stimulation; consequently, smaller devices can be developed. However, there is the disadvantage that selective stimulation of multiple muscles is difficult. Hence, implantable neurostimulators, which can conduct neuroprosthetic stimulation to generate selective contraction of multiple muscles, have been desired for FES applications. In the present study, we used wireless powering, and the receiver device was implanted inside a body to achieve implanted neuroprosthetic stimulation.

The control of limbs by the stimulation of multiple peripheral nerves with the combination of MISM leads to generate new treatments for patients who had peripheral nerve injury or neurodegenerative disease of peripheral nerves such as ALS. The precise control of stimulation current in different multiple peripheral nerves by one device is required to restore the functional motion of libs or legs. Conventionally, the motion generated by FES has not been precisely controlled, and most studies have involved an on/off mode of stimulation [25]. In order to achieve more precise motion control by FES, feedback, or feedforward control has been employed in recent studies [29,30]. These studies have shown better performance compared with conventional on/off motion, but they still have had limitations with respect to oscillation generation or precision of control. For example, Srinivasan et al. reported the closed-loop functional optogenetic stimulation (FOS) and FES of rat leg [29]. In their experiments, step response of a rat leg was 226 ms and 250 ms by FOS and FES, respectively. Our experimental result in the case of the target angle 40° showed similar response time 0.2 s (Figure 11a). The present study shows a fine level of controllability of a rat leg using visual feedback control. The device is wirelessly powered and could be implanted inside a body, which will decrease the risk of infection [24]. In the future, the receiver device will be miniaturized to an implantable size, and an implantable neurostimulator will be used to artificially generate the functional motion of hands or legs.

## 6. Conclusions

In this study, a wirelessly powered two-channel neurostimulator was developed to apply selective FES to the peroneal and tibial nerves in a rat, to generate flexion of the ankle joint. The neurostimulator used two coils to supply power wirelessly from a transmitter coil to a receiver coil by a magnetic resonance method. The receiver device, including a receiver coil, was connected to the peroneal and tibial nerves in a rat. The stimulation of the nerves was switched at the frequency delivered by the transmitter signal. Dorsal/plantar flexion of the rat ankle joint was selectively induced by the developed neurostimulator. The rat ankle joint angle was controlled by changing the stimulation electrode and current using a visual feedback control system. The rat ankle moved by following the target angle within an acceptable response time. The oscillation of the rat leg was reduced by modifying the P control.

## Supplementary Materials

The following are available online at https://www.mdpi.com/1424-8220/20/8/2210/s1, Videos related to step responses and visual feedback controls can be found in the supplemental video.

## References

[1] Ajiboye A.B., Willett F.R., Young D.R., Memberg W.D., Murphy B.A., Miller J.P., Walter B.L., Sweet J.A., Hoyen H.A., Keith M.W. (2017). Restoration of reaching and grasping movements through brain-controlled muscle stimulation in a person with tetraplegia: A proof-of-concept demonstration. Lancet.

[2] Bouton C.E., Shaikhouni A., Annetta N.V., Bockbrader M.A., Friedenberg D.A., Nielson D.M., Sharma G., Sederberg P.B., Glenn B.C., Mysiw W.J. (2016). Restoring cortical control of functional movement in a human with quadriplegia. Nature.

[3] Kurimoto S., Kato S., Nakano T., Yamamoto M., Takanobu N., Hirata H. (2016). Transplantation of embryonic motor neurons into peripheral nerve combined with functional electrical stimulation restores functional muscle activity in the rat sciatic nerve transection model. J. Tissue Eng. Regen. Med..

[4] Kato S., Kurimoto S., Nakano T., Yoneda H., Ishii H., Mita-Sugiura S., Hirata H. (2015). Successful Transplantation of Motoneurons into the Peripheral Nerve depends on the Number of Transplanted Cells. Nagoya J. Med. Sci..

[5] Ragnarsson K.T. (2007). Functional electrical stimulation after spinal cord injury: Current use, therapeutic effects and future directions. Spinal Cord.

[6] Deshmukh A., Brown L., Barbe M.F., Braverman A.S., Tiwari E., Hobson L., Shunmugam S., Armitage O., Hewage E., Ruggieri M.R. (2020). Fully implantable neural recording and stimulation interfaces: Peripheralnerve interface applications. J. Neurosci. Methods.

[7] Thakor N.V., Wang Q., Greenwald E. Bidirectional Peripheral Nerve Interface and Applications. Proceedings of the Engineering in Medicine and Biology Society, IEEE.

[8] Hügl S., Rülander K., Lenarz T., Majdani O., Rau T.S. (2018). Investigation of ultra-low insertion speeds in an inelastic artificial cochlear model using custom-made cochlear implant electrodes. Eur. Arch. Oto. Rhino. Laryngol..

[9] Kim Y., Kim J.S., Kim G.W. (2018). A Novel Frequency Selectivity Approach Based on Travelling Wave Propagation in Mechanoluminescence Basilar Membrane for Artificial Cochlea. Sci. Rep..

[10] Parastarfeizabadi M., Kouzani A.Z. (2017). Advances in closed-loop deep brain stimulation devices. J. Neuro Eng. Rehabil..

[11] Broccard F.D., Mullen T., Chi Y.M., Peterson D., Iversen J.R., Arnold M., Kreutz-Delgado K., Jung T.P., Makeig S., Poizner H. (2014). Closed-loop Brain-Machine-Body Interfaces for Noninvasive Rehabilitation of Movement Disorders. Annal. Biomed. Eng..

[12] Little S., Brown P. (2012). What brain signals are suitable for feedback control of deep brain stimulation in Parkinson’s disease?. Annal. N. Y. Acad. Sci..

[13] Kaniusas E., Kampusch S., Tittgemeyer M., Panetsos F., Gines R.F., Papa M., Kiss A., Podesser B., Cassara A.M., Tanghe E. (2019). Current Directions in the Auricular Vagus Nerve Stimulation II—An Engineering Perspective. Front. Neurosci..

[14] Romero-Ugalde H.M., Rolle V.L., Bonnet J.L., Henry C., Mabo P., Carrault G., Hernández A.I. (2018). Closed-loop vagus nerve stimulation based on state transition models. IEEE Trans. Biomed. Eng..

[15] Miyamoto T., Takeuchi M., Aoyama T., Nakano T., Kurimoto S., Hirata H., Hasegawa Y. Peripheral nerve stimulation device enabling adjustment of stimulation voltage. Proceedings of the International Symposium on Micro-NanoMechatronics and Human Science.

[16] Kiani M., Jow U.M., Ghovanloo M. (2011). Design and Optimization of a 3-Coil Inductive Link for Efficient Wireless Power Transmission. IEEE Trans. Biomed. Circuits Syst..

[17] Cho S.H., Xue N., Cauller L., Rosellini W., Lee J.B. (2013). A SU-8-Based Fully Integrated Biocompatible Inductively Powered Wireless Neurostimulator. J. Microelectromech. Syst..

[18] Navarro X., Krueger T.B., Lago N., Micera S., Stieglitz T., Dario P. (2005). Critical review of interfaces with the peripheral nervous system for the control of neuroprostheses and hybrid bionic systems. J. Peripher. Nerv. Syst..

[19] Shintake J., Sonar H., Piskarev E., Paik J., Floreano D. Soft pneumatic gelatin actuator for edible roborics. Proceedings of the IEEE/RSJ International Conference on Intelligent Robots and Systems.

[20] Cholleti E.R., Stringer J., Assadian M., Battmann V., Bowen C., Aw K. (2019). Highly Stretchable Capacitive Sensor with Printed Carbon Black Electrodes on Barium Titanate Elastomer Composite. Sensors.

[21] Thakur R., Nair A.R., Jin A., Fridman G.Y. Fabrication of a Self-Curling Cuff with a Soft, Ionically Conducting Neural Interface. Proceedings of the IEEE Engineering in Medicine and Biology Society.

[22] Peckham P.H., Kuntson J.S. (2005). Functional electrical stimulation for neuromuscular applications. Ann. Rev. Biomed. Eng..

[23] Sharma N., Stegath K., Gregory C.M., Dixon W.E. (2009). Nonlinear neuromuscular electrical stimulation tracking control of a human limb. IEEE Trans. Neural Syst. Rehabil. Eng..

[24] Ilfeld B.M., Gabriel R.A., Saulino M.F., Chae J., Peckham P.H., Grant S.A., Gilmore C.A., Donohue M.C., deBock M.G., Wongsarnpigoon A. (2017). Infection Rates of Electrical Leads Used for Percutaneous Neurostimulation of the Peripheral Nervous System. Pain Pract..

[25] Shon A., Chu J.U., Jung J., Kim H., Youn I. (2018). An implantable wireless neural interface system for simultaneous recording and stimulation of peripheral nerve with a single cuff electrode. Sensors.

[26] Jarc A.M., Berniker M., Tresch M.C. (2013). FES control of isometric Forces in the rat hindlimb using many muscles. IEEE Trans. Biomed. Eng..

[27] Schearer E.M., Liao Y.W., Perreault E.J., Tresch M.C., Memberg W.D., Kirsch R.F., Lynch K.M. (2016). Semiparametric identification of human arm dynamics for flexible control of a functional electrical stimulation neuroprosthesis. IEEE Trans. Neural Syst. Rehabil. Eng..

[28] Inmann A., Haugland M. (2004). Functional evaluation of natural sensory feedback incorporated in a hand grasp neuroprosthesis. Med. Eng. Phys..

[29] Srinivasan S.S., Mainmon B.E., Diaz M., Song H., Herr H.M. (2018). Closed-loop functional optogenetic stimulation. Nature Commun..

[30] Wolf D.N., Schearer E.M. Simple quasi-static control of functional electrical stimulation-driven reaching motions. Proceedings of the 9th International Conference on Neural Engineering.

